# Predictors of suboptimal glycemic control in people with type 2 diabetes and overweight or obesity: insights from baseline analysis of a diabetes-specific meal replacement study

**DOI:** 10.3389/fnut.2025.1696806

**Published:** 2026-01-12

**Authors:** Lew Leong Chen, Noorlaili Mohd Tohit, Arimi Fitri Mat Ludin, Zahara Abdul Manaf, Suzana Shahar

**Affiliations:** 1 Center for Healthy Ageing and Wellness, Universiti Kebangsaan Malaysia, Kuala Lumpur, Malaysia; 2 Biomedical Science Programme, Universiti Kebangsaan Malaysia, Kuala Lumpur, Malaysia; 3 Department of Family Medicine, Universiti Kebangsaan Malaysia Medical Centre (UKMMC), Kuala Lumpur, Malaysia; 4 Dietetic Programme, Universiti Kebangsaan Malaysia, Kuala Lumpur, Malaysia

**Keywords:** glycemic control, HbA1c, insulin resistance, obesity, type 2 diabetes

## Abstract

**Background:**

Individuals with obesity and type 2 diabetes (T2D) constitute a high-risk subgroup that frequently exhibits suboptimal glycemic control even under standard management strategies. This study aimed to characterize the baseline metabolic and sociodemographic profiles of participants enrolled in a randomized controlled trial evaluating the efficacy of a diabetes-specific meal replacement (DSMR), and to identify predictors of suboptimal glycemic control.

**Methods:**

A total of 156 adults with T2D and BMI ≥ 25 kg/m^2^ were recruited from a primary care clinic. Participants were randomized to receive either DSMR plus dietary consultation or dietary consultation alone for 12 weeks, followed by a 12-week monitoring period. Participants in treatment group replaced one meal per day for 5 days a week. Baseline assessments included anthropometric measurements, biochemical markers, dietary intake, physical activity, and quality of life. Pearson correlation and blockwise logistic regression were used to explore predictors of suboptimal glycemic control (HbA1c ≥ 8.0%). This study has obtained human ethics approval from RECUKM (JEP-2019-566) and is registered at the Thai Clinical Trials Registry (TCTR ID: TCTR20210921004).

**Results:**

The cohort had a mean age of 52.2 years and mean HbA1c of 8.52%, indicating suboptimal glycemic control. While no correlation was found between BMI and HbA1c, HOMA-IR and ALT were positively associated with suboptimal glycemic status. In the final regression model, HOMA-IR (OR = 1.099, *p* = 0.024), ALT (OR = 1.025, *p* = 0.022), and diabetes duration (OR = 1.119, *p* = 0.035) were significant predictors, while total protein and ALP were inversely associated.

**Conclusion:**

Elevated insulin resistance and liver-related markers were independent predictors of suboptimal glycemic control. The result underscores the importance of targeting metabolic dysfunction beyond weight alone in interventions for high-risk T2D populations. These findings also support the mechanistic rationale for targeted nutrition-based strategies aimed at improving insulin sensitivity and hepatic function. Moreover, they provide valuable context for interpreting participant responses within the ongoing trial. The DSMR used in this trial may offer a viable and sustainable option to improve glycemic control among high-risk individuals with T2D.

**Clinical trial registration:**

https://www.thaiclinicaltrials.org/show/TCTR20210921004, TCTR20210921004.

## 1 Introduction

Malaysia has one of the highest rates of diabetes in the Western Pacific region with a prevalence of 15.6%, which is significantly higher than neighboring countries such as Singapore (5.5%) and Indonesia (6.2%) (1–3). Individuals who are overweight or obese constitute a high-risk subgroup with a substantially greater likelihood of persistently suboptimal glycemic control despite standard management approaches (4, 5). Identifying the factors most strongly associated with elevated HbA1c can guide the development of targeted and efficient treatment strategies. This is particularly relevant in primary care settings, as resources can be allocated more efficiently and strategies designed to address individuals at greatest risk of persistent hyperglycemia.

Suboptimal glycemic control in T2D is influenced by a combination of non-modifiable factors such as age, sex and duration of diabetes, alongside modifiable determinants including obesity, insulin resistance, dyslipidemia, elevated liver enzymes and lifestyle behaviors. Addressing modifiable predictors is a key element of diabetes care as they provide opportunities for targeted intervention and long-term improvement in metabolic health. These insights align with key aspect of T2D management, where diabetes self-management and lifestyle modifications, including structured nutrition plans are used to address these modifiable risk factors and improve glycemic outcomes, particularly when tailored to an individual’s baseline risk profile (6).

In the present trial, a diabetes-specific meal replacement (DSMR) was incorporated as part of a partial meal replacement strategy alongside dietary consultation for participants with T2D who are overweight or obese. This manuscript describes a baseline cross-sectional analysis using pre-intervention data for the 156 enrolled participants, providing an overview of glycemic status, anthropometric measures and other metabolic indicators among adults with T2D receiving primary care in a local university hospital outpatient clinic at recruitment.

Predictors of suboptimal glycemic control, defined as HbA1c ≥ 8.0%, were examined to provide insights into the ongoing trial. This study is among the first in Malaysia to comprehensively examine metabolic, hepatic, and lifestyle predictors of suboptimal glycemic control in adults with type 2 diabetes and overweight or obesity. The findings can help provide new regional insights into the metabolic determinants of hyperglycemia and support the development of targeted nutrition-based strategies to improve diabetes management in high-risk populations. Identifying key predictors at baseline may improve understanding of differential responses to the intervention and guide the application of targeted DSMR strategies for Malaysia’s T2D population with overweight or obesity upon study completion.

## 2 Methodology

This study reports a baseline cross-sectional analysis of participants enrolled in a two-arm, randomized controlled clinical trial conducted at Universiti Kebangsaan Malaysia Medical Centre. Participant eligibility was determined based on predefined inclusion and exclusion criteria (Table 1), and electronic medical records were screened for potential participants. Sample size was determined using G*Power (version 3.1). An effect size of 0.06, derived from prior findings on mean HbA1c differences (primary outcome) was assumed. or a repeated-measures ANOVA (within–between interaction) with *α* = 0.05 and 80% power, and allowing for an anticipated 30% attrition rate, a total of 156 participants were recruited.

**Table 1:** Inclusion and exclusion criteria.

Inclusion criteria	Exclusion criteria
Aged 20–65 years old	On insulin treatment
Diagnosed with T2D for at least 6 months with baseline HbA1c levels between 7.5 and 12% for the past 3 months	With chronic kidney disease or on continuous ambulatory peritoneal dialysis or hemodialysis (GFR < 30 mL/min/1.73 m^2^).
Overweight or obese with BMI ≥ 25 kg/m^2^	With hepatic diseases (ALT > 120 IU/L)
On stable doses of any oral hypoglycaemic agents for the past 3 months	With history of chronic alcohol abuse
	Pregnant and lactating women
	Currently consuming any weight reduction products or any sliming prescriptions
	Currently involving in weight loss programs
	Record of COVID-19 diagnosis

**Figure 1:**
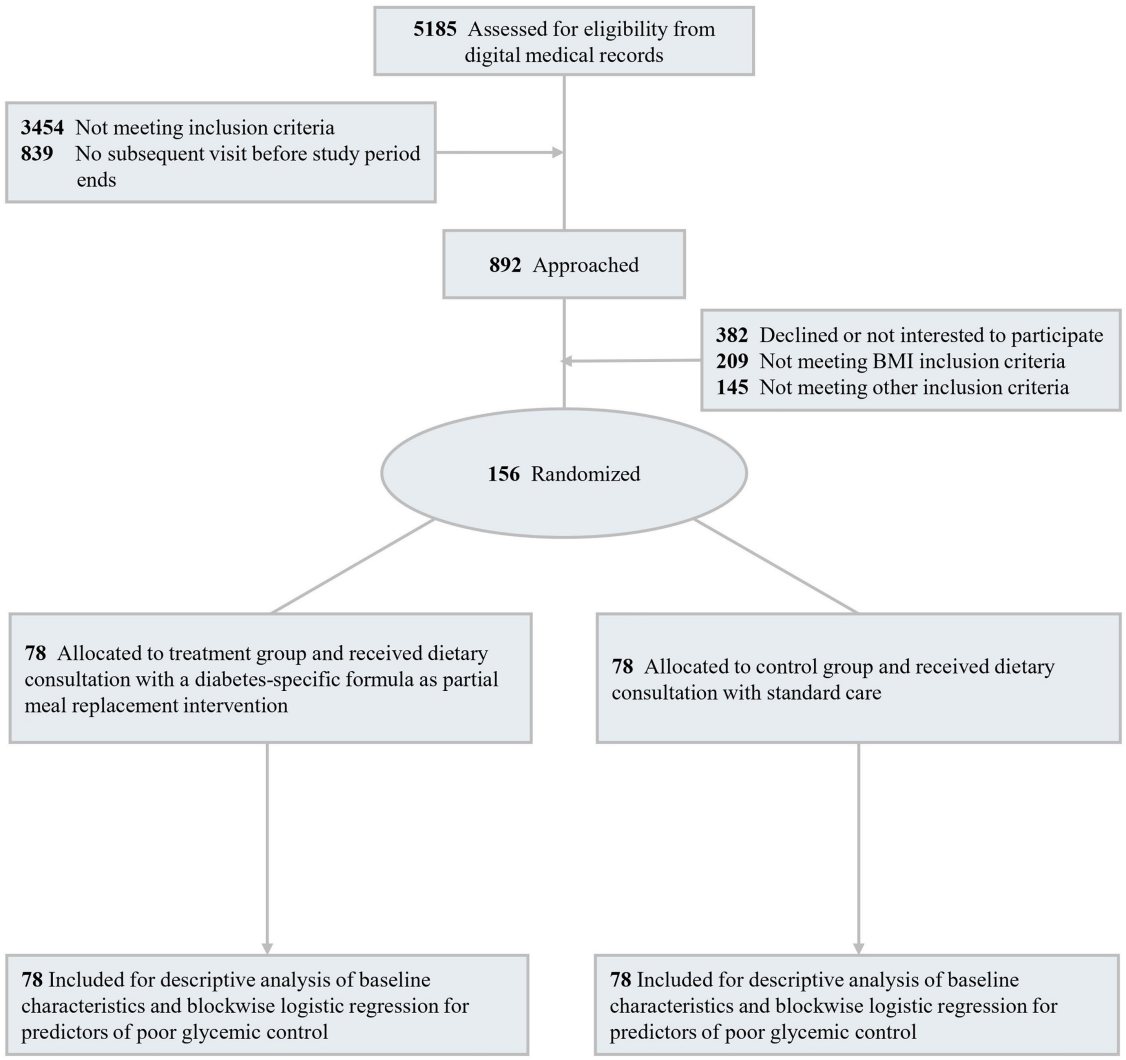
Research CONSORT flow diagram.

Eligible individuals who provided written informed consent were randomly assigned in a 1:1 ratio using minimization into either the treatment group, which received DSMR alongside standard dietary consultation, or the control group, which received dietary consultation alone. Randomization was performed using the WinPepi program with a minimization method (7). Allocation was balanced on sex (men, women), baseline HbA1c (≤ 8.0% vs. > 8.0%), and BMI (< 30 vs. ≥ 30 kg/m^2^). Due to the nature of the intervention, treatment allocation was not blinded to researchers or participants. To minimize selection bias, the randomization and statistical analysis was carried out by an independent statistician. Participants were informed of their assigned group before the intervention began. For the treatment group, DSMR was prescribed to replace one main meal a day, for 5 days a week. All participants continued to receive their routine care throughout the intervention period. Outcome assessments were conducted at baseline and at week 12, including clinical outcomes, sociodemographic data, dietary intake, and quality of life measured using validated structured questionnaires. Following the 12-week intervention phase, participants were monitored for an additional 12 weeks without further intervention, bringing the total study duration to 24 weeks. A CONSORT flow diagram outlining participant screening, eligibility, and enrollment for the baseline analysis is presented in Figure 1.

Ethical approval was granted by the Human Research Ethics Committee of Universiti Kebangsaan Malaysia (UKM) (Reference: JEP-2019-566), and the trial was prospectively registered with the Thai Clinical Trials Registry (TCTR20210921004). The detailed study protocol has been published previously (8). This report presents a baseline cross-sectional analysis of pre-intervention data with no intervention effects are evaluated herein.

### 2.1 Statistical analysis

Baseline characteristics were summarized using appropriate descriptive statistics. Continuous variables were assessed for normality and reported as means and standard deviations (SD), while categorical variables were presented as frequencies and percentages. Between-group comparisons (intervention vs. control) were conducted using independent-sample *t*-tests for continuous variables and Chi-square or Fisher’s exact tests for categorical variables, depending on cell size. Fisher’s exact test was used when any expected cell count was <5.

Variables including age, sex, ethnicity, education level, anthropometric measurements (weight, BMI, fat percentage, fat mass, visceral fat, muscle mass and waist-hip ratio), biochemical parameters (HbA1c, FPG, insulin, HOMA-IR, lipid, renal and liver profile), physical activity (total MET-minutes per week), and dietary intake (total energy and macronutrient distribution) were compared to assess baseline equivalence and characterize the cohort.

To explore the relationships between key metabolic parameters and glycemic status or adiposity, Pearson’s correlation was conducted using HbA1c and BMI as dependent variables. Independent continuous variables included FPG, fasting insulin, HOMA-IR, anthropometric measurements, lipid profile and blood pressure. This preliminary analysis informed the selection of predictors for subsequent multivariable logistic regression and helped screen for potential multicollinearity among variables.

A hierarchical binary logistic regression was conducted to identify predictors of suboptimal glycemic control, defined as HbA1c ≥ 8.0%. This cut-off was selected to identify a higher-risk subgroup, which aligns with the Malaysia Clinical Practice Guideline for T2D, with HbA1c targets of < 8.0% for selected adults with T2D. This is also consistent with targets used in conventional-therapy arms of major trials such as ACCORD and is supported by UKPDS evidence that microvascular risk rises markedly above 8.0% (9–11). The dependent variable was binary-coded (1 = HbA1c ≥ 8.0%, 0 = HbA1c < 8.0%). Prior to regression analysis, continuous predictors were screened for multicollinearity using linear regression diagnostics. Variables with tolerance < 0.10 or VIF > 5 were excluded or consolidated based on theoretical and statistical justification. A hierarchical block-entry approach was adopted to evaluate their incremental contribution to the model. Model 1 included non-modifiable sociodemographic variables: age, gender, race, working status, education level and diabetes duration. Model 2 incorporated modifiable clinical and metabolic indicators: BMI, fat percentage, visceral fat rating, HOMA-IR, triglycerides, low-density lipoprotein-cholesterol (LDL-C), high-density lipoprotein-cholesterol (HDL-C), albumin, total protein, bilirubin total, alanine aminotransferase (ALT) and alkaline phosphatase (ALP). Model 3 included diet intake and physical activity variables: total physical activity (MET-min/week), total caloric intake, and percentage of energy from carbohydrate.

Model performance was evaluated using Nagelkerke *R*^2^, and Hosmer–Lemeshow goodness-of-fit test. The overall predictive accuracy was assessed using the percentage of correctly classified cases. Adjusted odds ratios (ORs) with 95% confidence intervals (CIs) were reported for all predictors in the final model.

All statistical analyses were performed using IBM SPSS Statistics for Windows, Version 23.0 (IBM Corp., Armonk, NY, USA). Statistical significance was set at a two-tailed *p* value of < 0.05 for all comparisons.

## 3 Results

### 3.1 Baseline characteristics

The mean age of the study population was 52.2 ± 9.7 years (Table 2). Of the total sample, 81 participants (51.9%) were male and 75 (48.1%) were female. The cohort was predominantly Malay (75.0%), followed by Chinese (18.6%), Indian (5.8%), and other ethnicities (0.6%). More than half (50.6%) reported completing tertiary education, while 36.5% had secondary education and 12.8% had attained primary education or less. The majority of participants were currently employed (62.8%), with 23.1% retired and 14.1% unemployed. Self-reported alcohol consumption and smoking were low, with only 3.2% of participants reporting alcohol use and 7.7% reporting current smoking.

**Table 2:** Baseline characteristics of study participants.

Characteristics	Control (*n* = 78)	Treatment (*n* = 78)	Total (*n* = 156)	*p* value
Sociodemographic characteristics				
Age^a^	52.3 ± 9.4	52.1 ± 10.1	52.2 ± 9.7	0.922
Sex, *n* (%)^b^				
Male	39 (50.0)	42 (53.8)	81 (51.9)	0.631
Female	39 (50.0)	36 (46.2)	75 (48.1)	
Race, *n* (%)^b^				
Malay	58 (74.4)	59 (75.6)	117 (75.0)	0.764
Chinese	14 (17.9)	15 (19.2)	29 (18.6)	
Indian	5 (6.4)	4 (5.1)	9 (5.8)	
Others	1 (1.3)	0 (0.0)	1 (0.6)	
Educational status, *n* (%)^b^				
Primary education	9 (11.5)	11 (14.1)	20 (12.8)	0.096
Secondary education	35 (44.9)	22 (28.2)	57 (36.5)	
Tertiary education	34 (43.6)	45 (57.7)	79 (50.6)	
Education years^a^	12.3 ± 3.2	12.7 ± 3.3	12.5 ± 3.3	0.394
Working status, *n* (%)^b^				
Not working	11 (14.1)	11 (14.1)	22 (14.1)	0.927
Retired	17 (21.8)	19 (24.4)	36 (23.1)	
Currently working	50 (64.1)	48 (61.5)	98 (62.8)	
Current alcohol drinker, *n* (%)^b^	3 (3.8)	2 (2.6)	5 (3.2)	1.000
Current smoker, *n* (%)^b^	6 (7.7)	6 (7.7)	12 (7.7)	1.000
Presence of hypertension, *n* (%)^b^	51 (65.4)	58 (74.4)	109 (69.9)	
Duration of diabetes^a^	7.3 ± 4.3	6.9 ± 5.5	7.1 ± 4.9	0.639
Usage of antidiabetic drugs				
Metformin, *n* (%)^b^	74 (94.9)	74 (94.9)	148 (94.9)	1.000
DPP4 inhibitors, *n* (%)^b^	12 (15.4)	11 (14.1)	23 (14.7)	0.821
Sulfonylureas, *n* (%)^b^	56 (71.8)	60 (76.9)	116 (74.4)	0.463
SGLT2 inhibitors, *n* (%)^b^	15 (19.2)	22 (28.2)	37 (23.7)	0.188
GLP-1 receptor agonists, *n* (%)^b^	1 (1.3)	0 (0.0)	1 (0.6)	0.316
Thiazolidinediones, *n* (%)^b^	0 (0.0)	0 (0.0)	0 (0.0)	1.000
Glinides, *n* (%)^b^	0 (0.0)	0 (0.0)	0 (0.0)	1.000
Insulin, *n* (%)^b^	0 (0.0)	0 (0.0)	0 (0.0)	1.000
Biochemical profiles				
HbA1c (%)^a^	8.51 ± 0.96	8.53 ± 0.89	8.52 ± 0.92	0.904
HbA1c range, *n* (%)^b^				
≤8.0%	33 (42.3)	30 (38.5)	63 (40.4)	0.852
8.1–9.0%	24 (30.8)	27 (34.6)	51 (32.7)	
>9.0%	21 (26.9)	21 (26.9)	42 (26.9)	
FPG (mmol/L)^a^	8.61 ± 2.41	8.78 ± 2.43	8.70 ± 2.41	0.661
Insulin (mmol/L)^a^	20.01 ± 12.44	23.96 ± 17.99	21.99 ± 15.54	0.112
HOMA-IR^a^	7.75 ± 6.11	9.56 ± 8.48	8.65 ± 7.42	0.128
Triglyceride (mmol/L)^a^	1.71 ± 1.03	1.77 ± 0.94	1.74 ± 0.99	0.714
Total cholesterol (mmol/L)^a^	4.44 ± 1.18	4.58 ± 1.25	4.51 ± 1.21	0.471
HDL-C (mmol/L)^a^	1.10 ± 0.23	1.12 ± 0.24	1.11 ± 0.23	0.446
LDL-C (mmol/L)^a^	2.60 ± 1.03	2.72 ± 1.08	2.66 ± 1.05	0.463
Sodium (mmol/L)^a^	138.58 ± 2.70	139.12 ± 2.16	138.85 ± 2.46	0.172
Potassium (mmol/L)^a^	4.30 ± 0.45	4.33 ± 0.43	4.32 ± 0.44	0.703
Urea (mmol/L)^a^	4.29 ± 1.76	4.39 ± 1.47	4.34 ± 1.62	0.690
Creatinine (μmol/l)^a^	78.32 ± 27.80	80.67 ± 22.46	79.5 ± 25.21	0.562
eGFR^a^	86.06 ± 23.10	82.14 ± 20.28	84.1 ± 21.75	0.262
Albumin (g/l)^a^	40.74 ± 2.86	41.25 ± 3.27	41 ± 3.07	0.306
Total protein (g/l)^a^	75.85 ± 4.64	76.05 ± 4.27	75.95 ± 4.44	0.774
Bilirubin (μmol/l)^a^	13.38 ± 5.73	13.35 ± 6.45	13.37 ± 6.08	0.971
ALT (U/L)^a^	36.83 ± 20.78	45.81 ± 25.12	41.32 ± 23.42	**0.016** ^*^
ALP (U/L)^a^	84.28 ± 26.34	86.03 ± 30.05	85.15 ± 28.18	0.701
Anthropometric measurements				
Weight (kg)^a^	82.6 ± 16.1	83.1 ± 15.3	82.9 ± 15.6	0.836
BMI (kg/m^2^)^a^	31.2 ± 5.1	31.1 ± 4.2	31.2 ± 4.6	0.902
BMI category, *n* (%)^b^				
Overweight	36 (46.2)	37 (47.2)	73 (46.8)	0.873
Obesity	42 (53.8)	41 (52.6)	83 (53.2)	
Fat (%)^a^	38.3 ± 8.4	37.5 ± 8.2	37.9 ± 8.3	0.560
Fat mass (kg)^a^	31.9 ± 10.5	31.3 ± 9.2	31.6 ± 9.8	0.710
Muscle mass (kg)^a^	48.1 ± 10.6	48.9 ± 11.0	48.5 ± 10.8	0.660
Visceral fat rating^a^	14.1 ± 4.2	14.1 ± 4.2	14.1 ± 4.2	0.945
WC (cm)^a^	102.8 ± 8.7	103.6 ± 10.0	103.2 ± 9.3	0.571
WHR^a^	0.95 ± 0.05	0.95 ± 0.05	0.95 ± 0.05	0.773
Blood pressure				
SBP (mmHg)^a^	130 ± 12	130 ± 13	130 ± 13	0.712
DBP (mmHg)^a^	81 ± 8	82 ± 9	82 ± 9	0.429
Physical activity (MET min/week)^a^	1466.2 ± 2329.7	1415.7 ± 2270.9	1440.9 ± 2293.2	0.891
Dietary intake				
Energy (kcal/d)^a^	1521.16 ± 344.74	1495.04 ± 328.71	1508.1 ± 335.99	0.629
Carbohydrate (%)^a^	59.6 ± 6.6	57.7 ± 6.0	58.7 ± 6.4	0.162
Protein (%)^a^	20.9 ± 4.1	21.8 ± 3.7	21.3 ± 3.9	0.059
Total fat (%)^a^	19.5 ± 4.7	20.5 ± 3.7	20.0 ± 4.2	0.127
Quality of life scores				
ADDQOL score^a^	−3.75 ± 1.63	−3.91 ± 1.72	−3.83 ± 1.67	0.558
DDS score^a^	2.27 ± 0.62	2.36 ± 0.80	2.31 ± 0.72	0.430

^a^Data are presented as mean ± standard deviation. ^b^Data are shown as number (%) according to group total. HbA1c, Hemoglobin A1c; FPG, Fasting Plasma Glucose; HOMA-IR, Homeo-static model assessment of insulin resistance; BMI, Body Mass Index; WC, Waist circumference; WHR, Waist-Hip ratio; SBP, Systolic blood pressure; DBP, Diastolic blood pressure; HDL-C, High-Density Lipoprotein Cholesterol; LDL-C, Low-Density Lipoprotein Cholesterol; eGFR, estimated Glomerular Filtration Rate; ALT, Alanine Aminotransferase; ALP, Alkaline Phosphatase; DPP4, dipeptidyl peptidase-4; SGLT2, Sodium-Glucose Cotransporter 2; GLP-1, Glucagon-like Peptide-1; MET, Metabolic Equivalent of Task; ADDQOL, Audit of Diabetes-Dependent Quality of Life; DDS, Diabetes Distress Scale.

At baseline, participants presented with suboptimal glycemic control, as reflected by a mean HbA1c of 8.52 ± 0.92% and a mean fasting plasma glucose (FPG) of 8.70 ± 2.41 mmol/L. Additionally, the cohort had a mean diabetes duration of 7.1 ± 4.9 years. Anthropometric data indicated a mean body weight of 82.9 ± 15.6 kg and a mean BMI of 31.2 ± 4.6 kg/m^2^, categorizing the group as having class I obesity. Furthermore, elevated levels of fat percentage (37.9 ± 8.3%), fat mass (31.6 ± 9.8 kg), and visceral fat (14.1 ± 4.2) were observed, suggesting a high degree of adiposity and associated metabolic risk.

Lipid profiles were generally well managed, with mean triglycerides of 1.74 ± 0.99 mmol/L, total cholesterol of 4.51 ± 1.21 mmol/L, LDL-C of 2.66 ± 1.05 mmol/L, and HDL-C of 1.11 ± 0.23 mmol/L. Notably, 82% of participants were on statin therapy at baseline, which may have contributed to the favorable lipid control.

Renal and liver function profile were within normal clinical ranges, although ALT levels were significantly different between groups (*p* = 0.016), with higher values observed in the treatment group. Other biochemical markers, including insulin, HOMA-IR, sodium, potassium, urea, creatinine, and albumin, showed no significant baseline differences between groups.

At baseline, there were no significant differences observed between groups in total energy (1,508 ± 336 kcal/day), carbohydrate (58.7 ± 6.4%), protein (21.3 ± 3.9%), or fat intake (20.0 ± 4.2%). Physical activity levels, measured by total MET minutes per week, were also comparable between groups (mean 1440.9 ± 2293.2 MET-min/week).

Finally, baseline assessments of diabetes-related quality of life (ADDQoL) and diabetes distress (DDS) showed mean scores of −3.83 ± 1.67 and 2.31 ± 0.72, respectively, with no significant differences between groups.

### 3.2 Correlation of glycemic control and adiposity

Data analyses indicated that HbA1c was strongly and positively correlated with fasting plasma glucose (*r* = 0.532, *p* < 0.001) and moderately correlated with HOMA-IR (*r* = 0.371, *p* < 0.001) and fasting insulin (*r* = 0.188, *p* = 0.019), suggesting a close association between glycemic burden and underlying insulin resistance. Notably, no significant correlations were found between HbA1c and any anthropometric indices including BMI (*r* = 0.046, *p* = 0.571), fat percentage (*r* = −0.057, *p* = 0.483), or visceral fat rating (*r* = 0.090, *p* = 0.265) (Table 3).

**Table 3:** Correlation coefficients between HbA1c and BMI with fasting plasma glucose, fasting insulin, HOMA-IR, anthropometric measurements, lipid profile and blood pressure.

Variables	Statistic	FPG (mmol/l)	HbA1c	Fasting insulin	HOMA-IR	BMI (kg/m^2^)	Fat (%)	Fat mass (kg)	Visceral fat rating	WH ratio	TG (mmol/l)	TC (mmol/l)	HDL-C (mmol/l)	LDL-C (mmol/l)	SBP	DBP
HbA1c	*r*	**0.532** ^*^	–	**0.188** ^*^	**0.371** ^*^	0.046	−0.057	0.002	0.090	0.088	0.039	0.100	−0.008	0.100	−0.025	−0.020
	*p*	**<0.001**		**0.019**	**<0.001**	0.571	0.483	0.977	0.265	0.273	0.631	0.213	0.926	0.213	0.760	0.803
BMI	*r*	−0.038	0.046	**0.267** ^*^	**0.237** ^*^	–	**0.491** ^*^	**0.877** ^*^	**0.511** ^*^	0.006	0.031	−0.085	−0.148	−0.061	**0.358** ^*^	**0.186** ^*^
	*p*	0.636	0.571	**0.001**	**0.003**	–	**<0.001**	**<0.001**	**<0.001**	0.938	0.704	0.290	0.064	0.452	**<0.001**	**0.020**

Statistical significance using Pearson correlations at ^*^p < 0.05. HbA1c, glycated hemoglobin A1c; BMI, body mass index; HOMA-IR, homeostasis model assessment for insulin resistance; WH, Waist-Hip; HDL-C, High-density lipoprotein cholesterol; LDL-C, Low-density lipoprotein cholesterol; SBP, Systolic blood pressure; DBP, Diastolic Blood Pressure; r, Correlation coefficient.

BMI was significantly correlated with multiple measures of body composition, including fat percentage (*r* = 0.491, *p* < 0.001), fat mass (*r* = 0.877, *p* < 0.001), and visceral fat rating (*r* = 0.511, *p* < 0.001). Additionally, BMI showed significant positive correlations with fasting insulin (*r* = 0.267, *p* = 0.001) and HOMA-IR (*r* = 0.237, *p* = 0.003), indicating that greater adiposity was associated with increased insulin resistance. Modest positive associations were also observed between BMI and systolic (*r* = 0.358, *p* < 0.001) and diastolic (*r* = 0.186, *p* = 0.020) blood pressure.

### 3.3 Multivariable predictors of glycemic control

A blockwise logistic regression was conducted to identify baseline predictors of suboptimal glycemic control (defined as HbA1c ≥ 8.0%) among participants with type 2 diabetes (Table 4). Predictors were entered in three sequential blocks. The final model demonstrated acceptable overall fit, as indicated by a non-significant Hosmer–Lemeshow test (*χ*^2^ = 7.339, *p* = 0.501), a Nagelkerke *R*^2^ of 0.377, and a classification accuracy of 79.4%.

**Table 4:** Blockwise logistic regression predicting suboptimal glycemic control (HbA1c ≥ 8.0%).

Predictor	Model 1 OR (95% CI)	*p*	Model 2 OR (95% CI)	*p*	Model 3 OR (95% CI)	*p*
Age (years)	0.968 (0.921–1.018)	0.208	0.942 (0.877–1.012)	0.104	0.946 (0.879–1.018)	0.138
Gender	1.296 (0.624–2.694)	0.487	1.999 (0.255–15.652)	0.510	1.727 (0.198–15.083)	0.621
Ethnicity						
Chinese	0.948 (0.370–2.427)	0.911	0.572 (0.177–1.847)	0.350	0.403 (0.115–1.411)	0.155
Indian	5.161 (0.565–47.147)	0.146	4.272 (0.370–49.311)	0.245	4.764 (0.382–59.452)	0.225
Education level						
Primary	0.498 (0.136–1.824)	0.293	0.536 (0.114–2.525)	0.430	0.646 (0.127–3.298)	0.600
Secondary	1.103 (0.482–2.523)	0.816	1.709 (0.631–4.627)	0.292	1.598 (0.546–4.679)	0.392
Working status						
Not working	0.617 (0.201–1.891)	0.398	0.837 (0.231–3.031)	0.786	0.895 (0.230–3.482)	0.873
Retired	0.518 (0.189–1.420)	0.201	0.479 (0.149–1.543)	0.217	0.417 (0.127–1.371)	0.150
Diabetes duration	1.082 (0.992–1.179)	0.075	1.094 (0.991–1.207)	0.075	**1.119 (1.008–1.241)** ^*^	**0.035**
Anthropometric measurements						
BMI (kg/m^2^)			0.825 (0.610–1.114)	0.209	0.816 (0.601–1.107)	0.191
Fat %			1.088 (0.931–1.272)	0.291	1.098 (0.937–1.286)	0.250
Visceral fat			1.189 (0.854–1.657)	0.305	1.186 (0.845–1.665)	0.324
Biochemical profiles						
HOMA-IR			**1.098 (1.014–1.189)** ^*^	**0.021**	**1.099 (1.013–1.194)** ^*^	**0.024**
Triglycerides (mmol/L)			0.803 (0.504–1.279)	0.356	0.813 (0.510–1.296)	0.384
HDL-C (mmol/L)			0.985 (0.112–8.644)	0.989	1.013 (0.107–9.592)	0.991
LDL-C (mmol/L)			0.907 (0.592–1.391)	0.655	0.926 (0.597–1.436)	0.732
Albumin			0.956 (0.795–1.149)	0.629	0.961 (0.792–1.168)	0.691
Total protein			**0.846 (0.757–0.945)** ^*^	**0.003**	**0.830 (0.737–0.934)** ^*^	**0.002**
Bilirubin total			0.990 (0.923–1.062)	0.781	0.991 (0.924–1.064)	0.813
ALT			**1.023 (1.002–1.043)** ^*^	**0.029**	**1.025 (1.004–1.047)** ^*^	**0.022**
ALP			**0.980 (0.964–0.995)** ^*^	**0.012**	**0.979 (0.962–0.995)** ^*^	**0.011**
Physical activity and dietary intake						
MET (min/week)					1.000 (1.000–1.000)	0.845
Total Kcal					0.999 (0.998–1.001)	0.397
% Carbohydrate					0.948 (0.883–1.017)	0.134

Statistical significance using blockwise logistic regression test at ^*^p < 0.05. OR, Odds Ratio; CI, Confidence Interval; BMI, Body Mass Index; HOMA-IR, Homeostatic Model Assessment of Insulin Resistance; HDL-C, High-Density Lipoprotein Cholesterol; LDL-C, Low-Density Lipoprotein Cholesterol; ALT, Alanine Aminotransferase; ALP, Alkaline Phosphatase; MET, Metabolic Equivalent of Task; kcal, kilocalories. The reference categories for categorical variables were male (gender), Malay (ethnicity), primary education (education level) and currently working (working status). Model 1 included sociodemographic variables (age, gender, ethnicity, education level, working status, and diabetes duration). Model 2 added anthropometric and biochemical parameters (BMI, fat percentage, visceral fat, triglycerides, HDL-C, LDL-C, albumin, total protein, bilirubin, ALT, ALP, and HOMA-IR). Model 3 included dietary and lifestyle factors (total energy intake, percentage of carbohydrate intake, and physical activity in MET-min/week). Fasting plasma glucose (FPG), fat mass, waist-hip ratio, total cholesterol (TC), fasting insulin, and percentage intakes of protein and fat were excluded due to multicollinearity and model instability.

In model 1, none of the sociodemographic variables (age, gender, ethnicity, education level, working status, and diabetes duration) were statistically significant, though duration of diabetes indicated a possible association with suboptimal HbA1c control (OR = 1.082, 95% CI: 0.992–1.179, *p* = 0.075). Upon adding anthropometric and biochemical variables in model 2, the model’s predictive value improved. Several biochemical parameters emerged as statistically significant predictors of suboptimal glycemic control. HOMA-IR has shown a positive significant association with suboptimal glycemic control (OR = 1.098, 95% CI: 1.014–1.189, *p* = 0.021), indicating that higher insulin resistance was predictive of elevated HbA1c. Among liver-related markers, higher ALT was significantly associated with increased odds of suboptimal glycemic control (OR = 1.023, 95% CI: 1.002–1.043, *p* = 0.029). Conversely, higher total protein (OR = 0.846, 95% CI: 0.757–0.945, *p* = 0.003) and ALP (OR = 0.980, 95% CI: 0.964–0.995, *p* = 0.012) were associated with inversely associated with suboptimal control. Other anthropometric and lipid variables were not statistically significant in the adjusted model.

Model 3 introduced lifestyle and dietary intake variables, including total energy intake (kcal), percentage of carbohydrate intake, and total physical activity in MET-minutes per week. Notably, duration of diabetes became statistically significant in the final model (OR = 1.119, 95% CI: 1.008–1.241, *p* = 0.035), while HOMA-IR (OR = 1.099, 95% CI: 1.013–1.194, *p* = 0.024), total protein (OR = 0.830, 95% CI: 0.737–0.934, *p* = 0.002), ALT (OR = 1.025, 95% CI: 1.004–1.047, *p* = 0.022) and ALP (OR = 0.979, 95% CI: 0.962–0.995, *p* = 0.011) remained significant predictors. Dietary intake and physical activity variables were not statistically significant in the fully adjusted model.

## 4 Discussion

This prospective, randomized, controlled open label trial will be focusing on evaluating the effectiveness of a partial meal replacement intervention with DSMR on weight loss and glycemic control of participants with T2D and obese or overweight. Our study employed a real-world design to provide information on T2D management in a routine primary care setting. This study offers a detailed characterization of the baseline metabolic and demographic profiles of participants who were enrolled in the study.

Recruitment of participants into clinical trial has proven to be challenging. Poor recruitment can result in inadequate study numbers and underpowered trials (12). Our study was based on a primary care setting in a local university’s outpatient clinic in order to provide results generalizable to routine practice. Recruitment methods in our study were refined to include strategies such as phone contact, face to face meetings and incentives to help maximize recruitment. A total number of 156 participants was recruited to satisfy the power calculation and estimated dropout rate of 30%.

In this study, the amount of gender distribution (51.9% male and 48.9% female) for participants recruited was a close representative of national demographic data. Based on Department of Statistics Malaysia ratio of Malaysia’s male to female population in 2023 was 1.09:1 (13). Malaysia’s National Health and Morbidity Survey also showed similar percentage in prevalence of known diabetes among male and female (9.0% vs. 9.8%) (2). The mean age of participants recruited in this study (52.2 years) was also similar to the national average age at diagnosis for T2D in Malaysia, reported as 53 years in the National Diabetes Registry (14).

Glycemic control among the cohort was evenly distributed, providing a representative baseline for evaluating intervention impact. However, only 35.9% of the participants managed to maintain a well-controlled HbA1c level < 8.0% at the time of enrolment. This percentage slightly differed from statistics shown in Malaysia’s National Health and Morbidity Survey 2023 in which 56% of adult diabetes patients did not have good blood sugar control (3). The mean HbA1c was 8.5%, compared to 7.9% in the National Diabetes Registry, likely due to the trial’s inclusion criterion of HbA1c ≥ 7.5% (14).

When compared to baseline characteristics from other well-established clinical trial involving T2D patients, our cohort of participants had comparable BMI levels, consistent with obesity profiles reported in trials such as LOOK AHEAD (15), Direct (16), Why WAIT (17) and POWER (18). However, glycemic control in our cohort was notably poorer with mean HbA1c of 8.52%. Among these studies, only the ACCORD trial reported a comparable HbA1c at baseline (19). Some other trials have a lower baseline HbA1c level. In contrast, the DiRECT trial enrolled newly diagnosed patients with a lower mean HbA1c of 7.6%, while the Look AHEAD cohort had a longer diabetes duration but a lower average HbA1c of approximately 7.2%. Similarly, our participants also exhibited higher levels of insulin resistance. This comparison highlights that our trial population represents a metabolically high-risk group, with advanced insulin resistance and hyperglycemia. This underscores the trial’s relevance in addressing the needs of patients with suboptimal glycemic control despite being under standard care, a population whom may benefit more from targeted metabolic interventions and are often underrepresented in conventional lifestyle or dietary intervention trials.

Although our baseline result did not demonstrate a correlation between HbA1c and BMI, we found that BMI has significant correlation with fasting insulin and HOMA-IR. It is well established that excessive body weight and fat distribution impairs insulin secretion and induces insulin resistance (20). The obesity related mechanism towards insulin resistance might be due to several different pathways such as chronic inflammation, adipocyte dysfunction, oxidative stress, endoplasmic reticulum stress (ER stress), aging, hypoxia, and change in genetic makeup (21–24). A study by Wondmkun (25) stated that it was important to understand the impairment of insulin signaling which was related to obesity-induced diabetes to help discover better pharmacological treatment strategies and to help in prevention of obesity and T2D. These findings underscore the importance of assessing metabolic and hormonal profiles, such as HOMA-IR and liver-related biomarkers, rather than relying solely on anthropometric measures like BMI to stratify glycemic risk, particularly in intervention studies targeting overweight or obese individuals with T2D.

In this report, regression analysis supported this relationship, as HOMA-IR emerged as a consistent and independent predictor of suboptimal glycemic control (HbA1c ≥ 8.0%). Elevated HOMA-IR reflects increased insulin resistance, a central pathogenic mechanism in the development and progression of T2D, particularly among individuals with excess adiposity (26). Notably, the observed HOMA-IR values in this cohort were elevated compared to other major trials such as Look AHEAD and DiRECT, pointing to a metabolically high-risk phenotype. These findings suggest that insulin sensitization may also offer meaningful glycemic benefits, particularly in individuals with a more insulin-resistant metabolic phenotype such as observed in this cohort.

The use of DSMR in this trial reflects the growing interest in targeted nutritional interventions that extend beyond conventional calorie restriction to address specific metabolic disturbances in T2D and improve glycemic control (27). Understanding baseline characteristics and identifying independent predictors of suboptimal glycemic control will help to interpret the potential metabolic impact of the DSMR intervention, particularly given its low glycemic index and inclusion of cinnamon in its formulation, which has shown promise in improving insulin sensitivity and glucose metabolism (28).

The study also identified liver-related biochemical markers as relevant predictors. ALT, a marker of hepatic inflammation and steatosis, was positively associated with suboptimal glycemic status, consistent with previous studies linking elevated ALT to insulin resistance, non-alcoholic fatty liver disease (NAFLD) and increased hepatic gluconeogenesis (29, 30). Prior studies such as NHANES and Korean diabetes cohort studies have shown that elevated ALT is independently associated with HbA1c and incident diabetes (31, 32). In contrast, total protein and ALP were inversely associated with suboptimal glycemic control. While total protein may reflect nutritional or hepatic synthetic function, the mechanism underlying ALP’s inverse association remains unclear, possibly reflecting compensatory metabolic factors. These findings suggest a complex hepatic-metabolic contribution to glycemic regulation.

Notably, diabetes duration only achieved statistical significance in the fully adjusted model (Model 3), despite being non-significant in earlier models. This suggests a suppression effect whereby its impact was initially masked by more proximal metabolic markers such as HOMA-IR and liver profile. Previous studies have consistently shown that longer diabetes duration is associated with poorer glycemic control, largely due to progressive beta-cell dysfunction and reduced insulin secretory capacity (33, 34). Interestingly, the association shown here also raises the hypothesis that the adverse metabolic effects associated with prolonged disease duration may be modifiable. For instance, a meal replacement intervention using DSMR with potential insulin-sensitizing effects may attenuate the adverse glycemic burden associated with longer disease duration. While such a hypothesis cannot be directly tested from cross-sectional baseline data, it provides a mechanistic rationale for the intervention being evaluated in our trial.

### 4.1 Limitation

This is a cross-sectional analysis to report on the baseline characteristics of a cohort of participants in a meal replacement trial. Therefore, the causality on the relationship between the measured variables cannot be determined. We have employed self-reporting dietary records for dietary data collection which might lead to misreporting or recall bias. To mitigate this limitation, standardized diet records are being used to collect information for two weekdays, and one weekend to account for differences in intake throughout the week. Detailed instructions and measurements are also included and educated to the participants to help with accurate reporting of dietary intake. Next, the robustness of the prediction model was supported by good fit indices and classification accuracy. However, in the multivariable modeling process, several clinically relevant variables such as fasting insulin, total cholesterol, and macronutrient percentages for protein and fat were excluded from the final model due to multicollinearity and model instability. While this approach improved the reliability of the model, it may have narrowed the scope of the findings. Nonetheless, the retained predictors explained a meaningful proportion of variance in HbA1c levels. Future studies with larger sample sizes or alternative statistical methods could explore the combined effects of these related metabolic factors more effectively. Additionally, wide confidence intervals for certain categorical predictors, particularly ethnicity, indicated small subgroup sizes and increased variability, may have limited the precision of the estimated odds ratios. Although the model fit was adequate, these findings should be interpreted cautiously and validated in larger, more ethnically diverse samples. Lastly, while the study sample was representative of urban Malaysian T2D populations with overweight and obesity, generalizability to rural populations or other ethnic groups may be constrained.

## 5 Conclusion

This baseline analysis provides a comprehensive overview of the sociodemographic and metabolic profile of participants with T2D and overweight or obese enrolled in a randomized controlled trial investigating a partial meal replacement intervention with DSMR. The baseline characteristics between the two groups were identical and comparable to the gender ratio and age distribution of T2D population among Malaysia. This enhances the generalizability of subsequent findings related to glycemic control and weight management within similar settings. Importantly, baseline data revealed that insulin resistance and liver-related biomarkers were strong predictors of glycemic control. These findings suggest that, in addition to conventional weight-centric strategies, addressing underlying metabolic mechanisms such as insulin resistance may also be a valuable approach in improving glycemic outcomes. Although this analysis did not assess intervention effects, the findings provide baseline insights that support the rationale for evaluating nutrition-based interventions such as DSMR as a feasible and potentially sustainable dietary component within lifestyle-based strategies for adults with type 2 diabetes and overweight or obesity. These baseline insights will support the interpretation of post-intervention results and the design of nutrition-focused approaches aimed at improving glycemic control among high-risk clinical populations.

## Data Availability

The raw data supporting the conclusions of this article will be made available by the authors, without undue reservation.
